# Toward Spider Glue: Long Read Scaffolding for Extreme Length and Repetitious Silk Family Genes AgSp1 and AgSp2 with Insights into Functional Adaptation

**DOI:** 10.1534/g3.119.400065

**Published:** 2019-04-11

**Authors:** Sarah D. Stellwagen, Rebecca L. Renberg

**Affiliations:** * Department of Biological Sciences, University of Maryland Baltimore County, Baltimore, MD 21250; † General Technical Services, Adelphi, MD 20783

**Keywords:** spidroin, aggregate glue, long reads, full-length gene, spider silk, *A. trifasciata*

## Abstract

An individual orb weaving spider can spin up to seven different types of silk, each with unique functions and material properties. The capture spiral silk of classic two-dimensional aerial orb webs is coated with an amorphous glue that functions to retain prey that get caught in a web. This unique modified silk is partially comprised of spidroins (spider fibroins) encoded by two members of the silk gene family. The glue differs from solid silk fibers as it is a viscoelastic, amorphic, wet material that is responsive to environmental conditions. Most spidroins are encoded by extremely large, highly repetitive genes that cannot be sequenced using short read technology alone, as the repetitive regions are longer than read length. We sequenced for the first time the complete genomic Aggregate Spidroin 1 (AgSp1) and Aggregate Spidroin 2 (AgSp2) glue genes of orb weaving spider *Argiope trifasciata* using error-prone long reads to scaffold for high accuracy short reads. The massive coding sequences are 42,270 bp (AgSp1) and 20,526 bp (AgSp2) in length, the largest silk genes currently described. The majority of the predicted amino acid sequence of AgSp1 consists of two similar but distinct motifs that are repeated ∼40 times each, while AgSp2 contains ∼48 repetitions of an AgSp1-similar motif, interspersed by regions high in glutamine. Comparisons of AgSp repetitive motifs from orb web and cobweb spiders show regions of strict conservation followed by striking diversification. Glues from these two spider families have evolved contrasting material properties in adhesion (stickiness), extensibility (stretchiness), and elasticity (the ability of the material to resume its native shape), which we link to mechanisms established for related silk genes in the same family. Full-length aggregate spidroin sequences from diverse species with differing material characteristics will provide insights for designing tunable bio-inspired adhesives for a variety of unique purposes.

Spiders use a suite of remarkable silk and silk-derived materials for various applications during their life cycles, from wrapping prey and egg cases, to creating webs, lifelines, and prey capture glues. Individual orb weaving spiders, those that construct classic two-dimensional aerial capture webs, can have up to seven different silk types, each produced within specialized glands in the abdomen. Dragline silk, produced within the major ampullate glands, forms the frame of an orb web and is famous for its toughness comparable to that of steel (Vollrath and Knight 2001). The capture spiral threads of the web are made of flagelliform silk, produced in glands of the same name, which is highly extensible compared to other silks (Denny 1976). The sticky glue that forms droplets on the capture threads is composed of modified silk protein and other compounds produced within the aggregate glands, and functions to retain prey that get caught in a spider’s silken trap (Figure 1).

**Figure 1:**
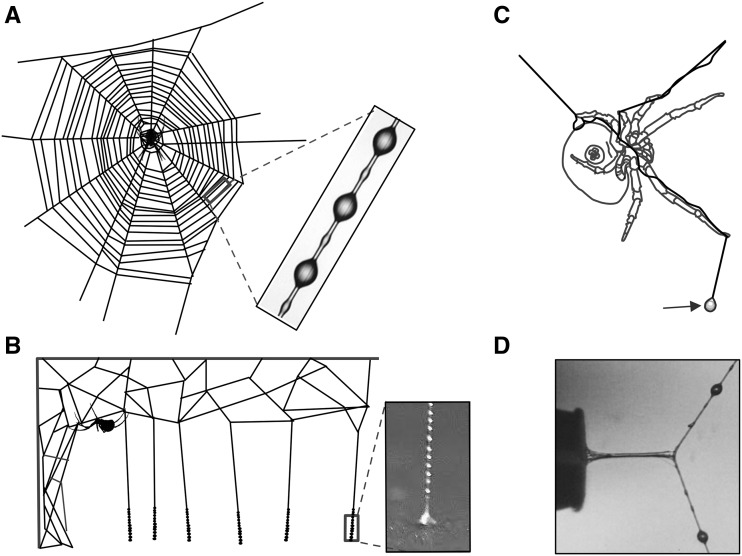
Aggregate spider glue from three spider types. (A) Orb weavers coat their web’s sticky capture spiral with glue, (B) cobweb weavers cover the lower portion of their triplines with glue to create ’gumfoot’ threads, (C) the bolas spider creates a large droplet of glue specialized for capturing moths, and (D) A stretching glue droplet after contacting a probe that was subsequently withdrawn at a constant rate. Inset images courtesy of Brent Opell.

In orb weavers, the capture glue originates within two pairs of aggregate glands that are connected by ducts to spigots located on spinnerets at the posterior end of the abdomen. Each pair of these semi-circular shaped glue spigots surrounds a flagelliform spigot, which produces the supporting thread as the glue is concurrently extruded (Sekiguchi 1952). The amorphous glue initially coats the flagelliform threads continuously, however as the two threads merge to form a single strand, hygroscopic low molecular mass compounds (LMMC) and salts within the glue absorb atmospheric moisture. Surface tension then causes the glue to separate into equally spaced droplets (Figure 1A)(Plateau 1873; Boys 1889; Edmonds and Vollrath 1992). A single droplet is composed of an aqueous solution containing adhesive glycoprotein (Tillinghast 1981; Opell and Hendricks 2010; Sahni *et al.* 2010) and the LMMC and salts that retain water and lubricate the glycoprotein (Fischer and Brander 1960; Anderson and Tillinghast 1980; Tillinghast and Christenson 1984; Townley *et al.* 1991; Opell *et al.* 2011, 2013; Sahni *et al.* 2014).

There is a variety of aggregate spider glue types, and each exhibits unique properties (Figure 1). Orb weaving spider glue acts as a viscoelastic solid, capitalizing on enhanced viscosity at more rapid extension rates and elasticity at slower extension rates (Sahni *et al.* 2010). This allows the glue to adhere to fast-moving insects at interception, and retain insects while a spider travels to and subdues them. Orb weaver glue responds instantly to humidity, which changes droplet volume, influences the performance of the glue, and is functionally optimized for a spider’s habitat (Opell *et al.* 2011; Sahni *et al.* 2011a; Stellwagen *et al.* 2014, 2015).

In contrast to orb weavers, cobweb weavers connect major ampullate silk threads from their webs to the ground, depositing aggregate glue on the lower portions to produce trip lines aimed at ambulatory prey (Figure 1B). The trip lines, or ’gumfoot’ threads, easily release from the ground, pulling insects into the air when accidentally intercepted, and the prey hang suspended until the spider can subdue them (Argintean *et al.* 2006). Unlike orb weaver glue, cobweb glue is considered a viscoelastic liquid, is resistant to changes in humidity, and is less extensible (Sahni *et al.* 2011a). Bolas spiders mimic female moth pheromones to attract their male counterparts, which they capture by swinging a single large glue droplet that hangs at the end of a silk strand (Figure 1C) (Eberhard 1977). The remarkable glue of these spiders is able to adhere in spite of a moth’s scales, which easily detach allowing them to escape other forms of predation (Sahni *et al.* 2011b; Diaz *et al.* 2018).

A fundamental understanding of silk function relies on knowledge of the protein sequences that compose the fibers, as the repetitive motifs that make up the bulk of a sequence influence the silk’s mechanical properties (Gosline *et al.* 1999). The primary proteins of spider silks are from the same family termed spidroins (‘spider fibroins’) (Hinman and Lewis 1992), and are often encoded by very long (>5kb), repetitive genes. The first full-length spidroin cDNAs were isolated from the orb weaving spider *Argiope bruennichi* (Scopoli,1772) in 2006 (Zhao *et al.* 2006). The ∼9 kb *A. bruennichi* cylindrical gland spidroins (CySp1 and CySp2; synonymous with tubiliform spidroins, TuSp), encode for silk that is used to wrap egg sacs. The gene that encodes for major ampullate (dragline) silk was fully sequenced from the black widow *Latrodectus hesperus* Chamberlin & Ivie (1935) genomic DNA the following year (Ayoub *et al.* 2007). To date, 19 large (>5kb), full-length, and classified spidroins (*i.e.*, assigned to a specific silk gland source) have been described (Table 1). Several other smaller or unclassified silk gland genes have also been characterized (Babb *et al.* 2017).

**Table 1:** Full-length, classified spidroins with >5 kb complete coding sequences (including start and stop codons), ordered from smallest to largest. Asterisk indicates a correction reported in Han and Nakagaki (2013) but not corrected on GenBank. CySp is synonymous with TuSp

Species	Spidroin	bp (cds)	Introns	Reference	Accession
*Araneus ventricosus*	MiSp	5298	Yes	Chen *et al.* (2012)	JX513956.1
*Araneus ventricosus*	TuSp1	5766	No	Wen *et al.* (2017)	MF192838.1
*Latrodectus hesperus*	MiSp_v1	6564	No	Vienneau-Hathaway *et al.* (2017)	KX584003.1
*Nephila clavipes*	MaSp-g	7398	Yes	Babb *et al.* (2017)	MWRG01015344.1
*Nephila clavipes*	TuSp	8631	No	Babb *et al.* (2017)	MWRG01000785.1
*Argiope bruennichi*	CySp1	8952	No	Zhao *et al.* (2006)	AB242144.1
*Latrodectus hesperus*	MaSp1	9390	No	Ayoub *et al.* (2007)	EF595246.1
*Argiope argentata*	TuSp1	9462	No	Chaw *et al.* (2018)	MF962652.2
*Nephila clavipes*	AcSp	9636	No	Babb *et al.* (2017)	MWRG01010975.1
*Argiope bruennichi*	CySp2*	9657	No	Zhao *et al.* (2006)	AB242145.1
*Nephila clavipes*	MaSp-h	10014	No	Babb *et al.* (2017)	MWRG01038219.1
*Argiope bruennichi*	MaSp2	10083	No	Zhang *et al.* (2013)	JX112872.1
*Araneus ventricosus*	AcSp	10338	No	Wen *et al.* (2018)	MG021196.1
*Nephila clavipes*	PiSp	10554	No	Babb *et al.* (2017)	MWRG01012117.1
*Latrodectus hesperus*	MaSp2	11340	No	Ayoub *et al.* (2007)	EF595245.1
*Argiope argentata*	AcSp1	13440	No	Chaw *et al.* (2014)	KJ206620.1
*Argiope argentata*	PySp1	17280	No	Chaw *et al.* (2017)	KY398016.1
*Nephila clavipes*	AgSp-d	17820	No	Babb *et al.* (2017)	MWRG01016148.1
*Latrodectus hesperus*	AcSp1	18999	No	Ayoub *et al.* (2013)	JX978171.1

Initial evidence for aggregate glue gene sequences was derived from what were thought to be two full-length transcripts encoding the glue proteins of orb weaver *Nephila clavipes* (Linnaeus,1767), and were named ASG1 (Aggregate Spider Glue; 1,221 bp) and ASG2 (2,145 bp; Choresh *et al.* (2009)). Later it was determined that ASG1 expression is not unique to aggregate glands, and that the ASG2 sequence was incomplete (Collin *et al.* 2016). Collin *et al.* (2016) also extended the known sequence to 2,821 bp, and discovered that the predicted ASG2 C-terminus aligns with other spidroins, renaming it AgSp1 (Aggregate Spidroin 1) to follow gene family convention, which we have adopted here. During sequencing of the first orb weaving spider genome, which focused on describing spidroins from *N. clavipes*, the 5′ sequence for AgSp1 was discovered (Babb *et al.* (2017); called AgSp-c, following the study’s own spidroin naming scheme), and the known length extended to at least 11,106 bp. This study further reported three other aggregate gland spidroins (AgSp-a, AgSp-b, and AgSp-d).

We combined short read Illumina and long read Oxford Nanopore technologies to sequence aggregate spidroins from orb weaving spider *Argiope trifasciata* (Forskål 1775), a large cosmopolitan species that is abundant in our location, and which has had many aspects of its biology extensively researched. Furthermore, we analyzed aggregate gland transcript data from three orb weavers and four cobweb weavers to discover and compare the repetitive motifs of aggregate spidroins. Similar to solid silks, understanding the underlying mechanisms that provide aggregate spider glue with its diverse properties will provide insight into designing novel bio-inspired adhesives (Malay *et al.* 2017; Teulé *et al.* 2012; Adrianos *et al.* 2013). Synthetic spider glues would not require replicating the spinning process that transforms liquid protein dope into solid threads within a spider’s gland duct, traits that have made synthetic silks challenging to scale. The aggregate glue is extruded without the same processing as fibers (Townley and Tillinghast 2013), and synthetic versions could provide water-soluble and biodegradable solutions to problems ranging from pest control to temporary sealants.

We hypothesize that differences in orb and cobweb glue material properties will be reflected in the predicted protein backbone of aggregate spidroins, as inferred from mechanisms established for related silks. Spider glue droplets exhibit 1) adhesion, typically to insect prey, 2) extensibility, to remain in contact with struggling prey, and 3) elasticity, to retain prey after extension. Glue adhesion has been attributed to glycosylation (Singla *et al.* 2018), however potential mechanisms for extensibility and elasticity have only been described for other silks. Orb web weaver glue droplets are more extensible (*i.e.*, stretch farther, Figure 1D) than cobweb weaver droplets (Sahni *et al.* 2011a), and we expect to find sequence similarity related to increased extensibility in paralogous genes, such as glycine-proline-glycine motifs found in minor ampullate silks (Vienneau-Hathaway *et al.* 2017), to be more prominent in orb weaver glue. Elasticity, or the ability of a material to resume its original shape, is an important property for spider glues. Elasticity causes glue droplets to return to their original shape, retaining prey in the web after interception. We expect aggregate spidroins to contain sequence that may contribute to the elastic properties of the predicted protein, such as hydrophobic residues, and we expect these characteristics to be found across species.

## Materials and Methods

### Specimen collection and dissection

Adult female *A. aurantia* and *A. trifasciata* were collected from Schooley Mill Park in Highland, Maryland during September 2016 and August 2017. Specimens were kept in cages and allowed to build webs overnight. After building a fresh capture spiral in the morning, specimens were killed and dissected in Invitrogen RNALater (cat.no. AM7021) before 1200h. Aggregate and major ampullate glands, as well as fat body tissue samples were collected and stored in RNALater at -20${}^{\circ }$.

### Illumina RNA sequencing and assembly

Twenty-four RNA samples were extracted for Illumina sequencing from the following tissues: aggregate glands from 6 individuals of each species, major ampullate glands from 3 individuals of each species and fat body tissue from 3 individuals of each species. Total RNA was extracted according to the Qiagen miRNeasy protocol (cat.no. 217004) including the optional on column DNAse digestion. Quality and quantity of total RNA was assessed using the DeNovix DS-11+ Spectrophotometer (Denovix, Wilmington, DE, USA) and Qubit 3.0 Fluorometer (ThermoFisher Scientific, Waltham, MA, USA), which met the criteria for library preparation. Purified total RNA was prepared for sequencing using the Illumina TruSeq Stranded mRNA library prep kit (cat.no. RS-122-2101), which includes an mRNA selection step, and run in-house on a NextSeq500 using a High Output kit with 2 × 150 PE cycles on October 31, 2016.

Sequence data sets obtained from this study and publicly available online data sets (Supplementary Table S1; http://www.ncbi.nlm.nih.gov/sra) were trimmed and filtered for quality using Trimmomatic v.0.36 (Bolger *et al.* 2014) and *de novo* assembled using Trinity (Grabherr *et al.* 2011) set with default parameters. Resultant contigs were searched to select any sequences that contained similar regions and motifs to aggregate spidroins previously published.

### Oxford Nanopore RNA Sequencing

Messenger RNA was extracted and pooled using the NEB Magnetic mRNA Isolation Kit (cat.no. S1550S) from 3 adult female *A. trifasciata* aggregate glands. The mRNA was then prepared for Oxford Nanopore sequencing using the Direct RNA Sequencing Kit (cat.no. SQK-RNA001). We replaced the recommended RT and buffer with those from the Thermo Scientific Maxima H Minus First Strand cDNA Synthesis kit (cat.no. K1681). The cDNA produced during library preparation is not sequenced, but provides stability for direct sequencing of the RNA molecules. Samples were run on a SpotON Flow Cell (R9.5; cat.no. FLO-MIN107), and resultant fast5 files were basecalled using Oxford Nanopore’s program Albacore v2.1.3.

### Oxford Nanopore gDNA Sequencing

Two juvenile *A. trifasciata* were collected from Schooley Mill park on July 23, 2018 and kept overnight in containers. High molecular weight DNA was gently extracted and pooled from whole, fresh specimens the next day using the MasterPure Complete DNA and RNA Purification Kit following the DNA Purification section protocol (cat.no. MC89010). A total of 10 µg of the gDNA extraction (pooled from both individuals) was loaded onto a Sage Science BluePippin cassette (cat.no. BLF7150) and run with a 20 kb high pass threshold overnight. The resultant elution was used directly in Oxford Nanopore’s 1D Genomic DNA by Ligation protocol (SQK-LSK109). A total of four runs were completed using SpotON Flow Cells (R9.4; cat.no. FLO-MIN106) and resultant fast5 files were basecalled using Oxford Nanopore’s program Albacore v2.2.7.

### Analyses

Sequence alignment and analysis was conducted using Geneious v11.0.5 (Kearse *et al.* 2012) with the Geneious alignment and ClustalW tools (Thompson *et al.* 1994). Maximum likelihood phylogenetic analyses were performed using the RAxML v8.2.11 Geneious plugin (Stamatakis 2014) with the GTRGAMMA model with 10,000 bootstrap replicates. RSEM (Li and Dewey 2011), EdgeR (Robinson *et al.* 2010), and R (R Core Team 2014) were used for differential gene expression analyses. Statistical significance of amino acid percentages and TPM comparisons was calculated using Student’s *t*-tests. Hydrophobicity was predicted using the Kyte and Doolittle scale from online Expasy tools and a window size of 3.

### Data Availability

The authors affirm that all data necessary for confirming the conclusions of this article are represented fully within the article, its tables, figures, and supplemental material. Supplemental material available at FigShare: https://doi.org/10.25387/g3.7949351.

## Results

**Figure 2:**
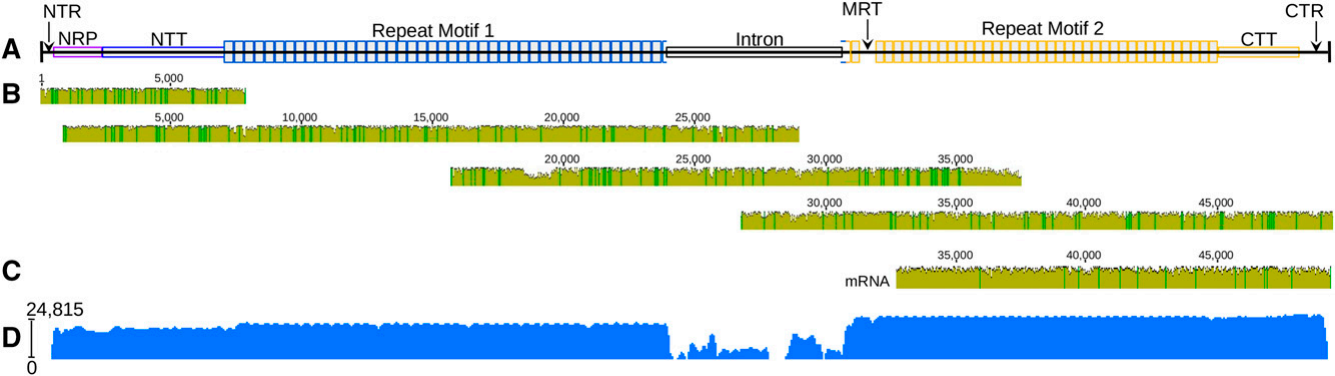
*A. trifasciata* Aggregate Spidroin 1 (AgSp1) schematic, aligned Oxford Nanopore gDNA reads, longest alignable mRNA read, and mapped read coverage. (A) AgSp1 consists of 42,270 bp of coding sequence and ∼6,690 bp of intronic sequence, totalling ∼48,960 bp of genomic sequence (intronic sequence could not be corrected with short reads derived from mRNA). Abbreviations correspond to regions of the predicted protein: NTR = N-terminal region; NRP = N-terminal repeats; NTT = N-terminal transition; MRT = mid-repeat transition; CTT = C-terminal transition, CTR = C-terminal region. (B) Individual alignment of four Oxford Nanopore reads to the consensus AgSp1 together cover the entirety of the gene. (C) Alignment of a 16.4 kb mRNA transcript to the consensus AgSp1. (D) Log read coverage of Illumina RNAseq data generated from aggregate gland tissue mapped to AgSp1.

We sequenced, assembled, and corrected two highly expressed spidroin genes from the aggregate glands of orb weaving spider species *Argiope trifasciata*. To achieve full-length sequences, we used high-accuracy RNAseq short reads from Illumina to correct error-prone gDNA long reads from Oxford Nanopore. Aggregate Spidroin 1 (AgSp1) is encoded by 42,270 bp from two similarly sized exons spanning ∼48,960 bp of genomic DNA, which includes a single ∼6,690 bp central intron (Figure 2 and Supplementary Fig. S1; accession#: MK138561). AgSp2 is encoded by 20,526 bp from a short 5′ and large 3′ exon, and contains an ∼31,455 bp intron, in total spanning ∼51,981 bp of gDNA (Figure 4 and Supplementary Fig. S2; accession#: MK138559). We collected RNAseq short reads for long read correction, resulting in highly accurate coding sequences for these two genes, however apart from a few reads from pre-mRNA, RNAseq reads do not cover genomic intron sequence and is therefore uncorrected. Long read scaffolds and pre-mRNA reads did allow identification of putative splice sites for both aggregate spidroins (Supplementary Fig. S3).

AgSp1 and AgSp2 are members of the spidroin family (Collin *et al.* 2016) and are encoded by the same general N-terminus/Repeat(n)/C-terminus pattern found in this family, however the size and internal organization are distinct. We have broken the predicted protein of each spidroin into several regions. AgSp1 regions include: 1) an N-terminal region (NTR), 2) a short region of N-terminal repeats (NRP), 3) an N-terminal transition (NTT, region with degenerate, repeat-similar structure), 4) 43 iterations of repeat motif 1 (RM1), 5) 38 iterations of repeat motif 2 (RM2), 6) a C-terminal transition (CTT, region with degenerate, repeat-similar structure), and 7) a C-terminal region (CTR) (Figure 2 and Supplementary Fig. S1). AgSp2 consists of an NTR and CTR flanking areas rich in glutamine (QRR - glutamine-rich region) that intersperse variable sized blocks of an iterated single repeat motif (RM) (Figure 4 and Supplementary Fig. S2).

Current RNA sequencing technology does not provide reliable full-length sequencing of extreme-sized transcripts, however we were able to sequence a ∼16.5 kb continuous RNA read of each aggregate spidroin using Oxford Nanopore’s MinION and direct RNA sequencing kit (Figures 2C and 4C). These enormous reads terminated with their 5′ ends still in the repetitive regions a few iterations before the genes’ respective introns. While most classified spidroins >5 kb in length are single exon (Table 1), AgSp1 and AgSp2 each have a large, single intron (Figures 2 and 4).

### AgSp1

The 5′ end of AgSp1 encodes for the N-terminal region, which includes the N-terminal domain that is conserved across spidroins (Spidroin_N domain superfamily BLASTx e-value 3.36e-14), as well as linker sequence. Following the NTR, AgSp1 contains a short, 1,758 bp region that consists of translated TGSYITGESGSYD repetitions before connecting to the N-terminal transition encoding region. The N- and C- terminal transition regions that flank the iterative central repeat motif blocks (discussed below), consist of sequence that is unique enough to be assembled using only short reads, however the amino acid translation clearly resembles and aligns with the repeat motifs. These degenerate repeats become increasingly less conserved as the sequence approaches either terminus (Supplementary Fig. S1). The C-terminal transition region is shorter than the N-terminal transition, and maintains the same organization as the internal repeats, with the majority of variation occurring toward the end of each repeat iteration. The C-terminal region, including linker sequence and conserved domain, has been previously described (Choresh *et al.* 2009; Collin *et al.* 2016).

**Figure 3:**
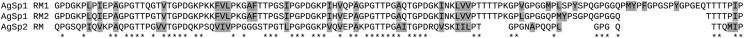
Predicted amino acid sequence of repeat motif 1 (RM1) and repeat motif 2 (RM2) from AgSp1 aligned with the predicted amino acid sequence of the repeat motif (RM) from AgSp2 of *A. trifasciata*. Conserved residues are marked with an asterisk below, and hydrophobic residues are highlighted.

The central repetitive region of AgSp1 contains two distinct repeat motifs. There are 43 iterations of repeat motif 1 (RM1, 387 bp) and 38 iterations of repeat motif 2 (RM2, 339 bp); RM1 and RM2 predicted amino acid sequences are mostly conserved except for a few short regions (Figure 3). These results demonstrate that previously reported AgSp1 repeat motifs were representative of a portion the C-terminal transition region, rather than the dominant repeat motifs described here (Supplementary Fig. S1, underlined sequence in the CTT aligns to reported repeat of *Argiope argentata* (Fabricius, 1775) from Collin *et al.* (2016)).

In addition to *A. trifasciata*, we *de novo* assembled Illumina RNAseq data from the aggregate glands of *Argiope aurantia* Lucas (1833), and assembled publicly available aggregate gland RNAseq data sets from GenBank to discover the repeat motifs of aggregate spidroin genes from several other species: an additional orb weaver, *N. clavipes*, and three cobweb weavers, *Steatoda grossa* (C. L. Koch 1838), *Latrodectus hesperus*, and *Latrodectus geometricus* Koch (1841) (SRR accession numbers in Supplementary Table S1). AgSp1 from a fourth cobweb species, *Parasteatoda tepidariorum* (C. L. Koch 1841), was discovered after a BLASTp search using AgSp1 RM2 as a query (∼14.8 kb; accession#: XM_021148663.1). This sequence was assembled using short read data and is likely incomplete, though many repeat motifs are present and therefore included in our comparison.

Across species, repeat motifs can be classified into four subgroups with many conserved residues and completely conserved lengths, followed by a variable ’tail’ (Figure 6). Subgroups from all species begin with a GPXG group preceding a high proportion of hydrophobic amino acids (Figure 6A, highlight). The first three subgroups contain regions high in serine/threonine residues, which are likely glycosylated, and are flanked by proline and glycine. The AgSp1 repeat tail region of orb weaving species is distinct from the cobweb species, which is both longer and contains stretches of poly-threonine on either end. The tail regions also contain GGQ, PGG, GPG, and QGP motifs found frequently in other spidroins, and the tail of *A. trifasciata* aligns well to repeats of major ampullate spidroin 2 (MaSp2; Figure 6B, highlight). All species have several variations of their repeats, however the subgroups remain conserved with most variation again lying in the tail region (Supplementary Figure S4). Without full length sequences from the other species, it is difficult to assess if these repeat motifs are part of transitional regions or if dominating motifs are present as in *A. trifasciata*. However, the motifs chosen for comparison were the most similar to repeat motifs of the *Argiope* species.

Percentages of the most prominent or glycosylation-associated amino acids that make up the AgSp1 glue repeats vary between orb web weavers and cobweb weavers (Figure 6A, Table 2). The percent of glycine is similar between the repeats motifs of the two glue types (∼22%, *P* = 0.7847), however orb web repeats have a higher percent of proline than cobweb repeats (23.1% *vs.* 19.6%, respectively, *P* < 0.0001). The total amount of potentially O-glycosylated residues (serine + threonine) is the same between the two glue type repeats (17.8%), however orb web repeats contain more threonine than cobweb repeats (14.9% *vs.* 10.4%, respectively, *P* = 0.0003) and cobweb repeats contain more serine than orb web repeats (7.3% *vs.* 2.3%, respectively, *P* = 0.0006).

**Table 2:** Percentage of glycosylated and glycosylation-associated amino acids (aa) in the repeat motifs of three orb web and four cobweb species. Bold indicates significant differences (*P* < 0.5)

	orb weavers			cobweb weavers				average	
aa	*Ncl*	*Atr*	*Aau*	*Pte*	*Sgr*	*Lhe*	*Lge*	*orb*	*cob*
**G**	24.1	21.2	21.2	20	25	20.8	20.8	22.2	21.7
**P**	23.3	23	23	18.9	19.8	19.8	19.8	**23.1**	**19.6**
**T**	14.7	15	15	10.5	10.4	9.4	11.5	**14.9**	**10.4**
**S**	3.4	1.8	1.8	6.3	7.3	8.3	7.3	**2.3**	**7.3**
**S+T**	18.1	16.8	16.8	16.8	17.7	17.7	18.8	17.2	17.8

We conducted maximum likelihood analyses of AgSp1 repetitive motifs for the three orb web and four cobweb weaving species rooted with the AgSp2 repeat from *A. trifasciata* (Figure 6C). Bootstrap support for phylogenetic analysis reflects the most recent described spider relationships (Garrison *et al.* 2016; Bond *et al.* 2014; Fernández *et al.* 2014), as well as analyses based on the AgSp1 carboxyl-terminus (Collin *et al.* 2016).

### AgSp2

**Figure 4:**
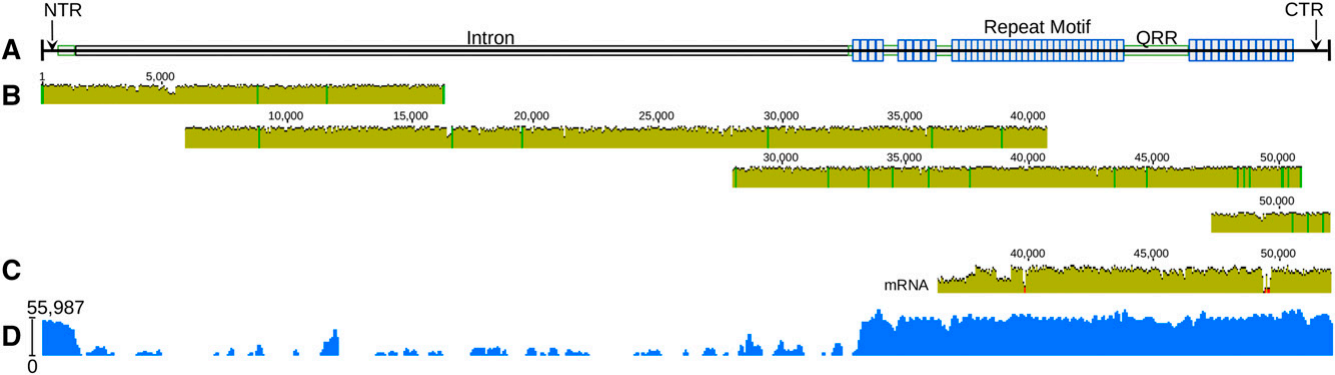
*A. trifasciata* Aggregate Spidroin 2 (AgSp2) schematic, aligned Oxford Nanopore gDNA reads, longest alignable mRNA read, and mapped read coverage. (A) AgSp2 consists of 20,526 bp of coding sequence and ∼31,455 bp of intronic sequence, totalling ∼51,981bp of genomic sequence (intronic sequence could not be corrected with short reads derived from mRNA). Abbreviations correspond to regions of the predicted protein: NTR = N-terminal region; QRR = glutamine-rich region (all regions in green); CTR = C-terminal region. (B) Individual alignment of four Oxford Nanopore reads to the consensus AgSp2 together cover the entirety of the gene. (C) Alignment of a 16.5 kb RNA transcript to the consensus AgSp2. (D) Log read coverage of Illumina RNASeq data generated from aggregate gland tissue mapped to AgSp2.

AgSp2 is half the size of AgSp1 and is organizationally distinct. The 5′ end also contains sequence that belongs to the the Spidroin_N domain superfamily (BLASTx e-value 3.49e-13), however it lacks the subsequent N-terminal repeats found in AgSp1. Instead, the N-terminal region leads directly into a short glutamine-rich region, and similar regions intersperse iterated AgSp2 repeat motif blocks along the length of the gene. AgSp2 has one main repeat motif (RM), which forms two main blocks of 14 and 27 iterations, as well as several smaller blocks (Figure 4; Supplementary Fig. S2). As in AgSp1, there are repeat motifs that appear to be degenerate, and are usually located at ends of repeat blocks. The repeat motif of AgSp2 is similar to those of AgSp1 (Figure 3), with four conserved subgroups and a unique tail. AgSp2 corresponds to AgSp-b of *N. clavipes* reported in Babb *et al.* (2017), however we did not find evidence for aggregate spidroins AgSp-a or AgSp-d from their report.

We identified the putative AgSp2 from cobweb weavers *L. geometricus* (accession#: MK138562) and *L. hesperus* (accession#: MK138563; Supplementary Fig. S5) after *de novo* assembly of online data sets (Supplementary Table S1). The assembly software was able to completely reconstruct AgSp2 in both species, with no evidence of truncation as seen in AgSp1. AgSp2 is extremely reduced to less than 2 kb in these species, and has lost the repetitious nature of most spidroins. BLASTp results identify these genes as flagelliform (FLAG) spidroin N-termini, however further analysis suggests they have been misidentified. Phylogenetic analysis of N-termini from cobweb AgSp2, *A. trifasciata* AgSp1 and AgSp2, and *A. argentata* FLAG results in cobweb AgSp2 grouping with *A. trifasciata* AgSp2, which are sister to *A. trifasciata* AgSp1 to the exclusion of FLAG (Supplementary Fig. S6). Furthermore, normalized FPKM expression data from Clarke *et al.* (2017) show that AgSp2 transcripts (transcript IDs: *L. geometricus* Lg3tf002378g0u and *L. hesperus* Lh3tf002378g0u) are highly expressed in the anterior aggregate glands relative to the other gland and body tissues (Gene Expression Omnibus accession#: GSE95367).

### Gene Expression Analyses

**Figure 5:**
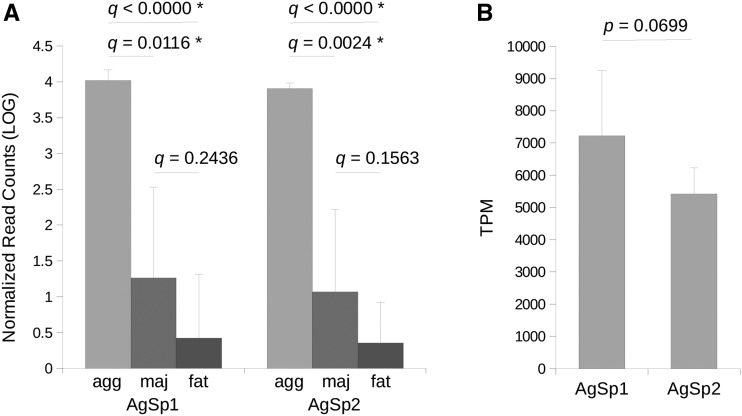
Gene expression analyses for AgSp1 and AgSp2 of *A. trifasciata*. Genes were each manually edited to 999 bp of the 3′ ends for analyses. (A) Log number of normalized reads that align to AgSp1 and AgSp2 from aggregate gland tissue (agg), major ampullate gland tissue (maj) and fat body tissue (fat). Line ends correspond to statistical comparisons between tissues. (B) Transcripts per million (TPM) for AgSp1 and AgSp2 averaged across six aggregate gland tissue samples. Asterisks indicate significant q- and p-values (<0.05).

We performed gene expression analyses using transcript data sequenced from *A. trifasciata* aggregate gland tissue to compare AgSp1 and AgSp2 expression, and from major ampullate silk gland and fat body tissues as controls. AgSp1 and AgSp2 are among the most highly expressed genes within the aggregate glands, with significantly (q < 0.05) less expression detected in the major ampullate or fat body tissues (Figure 5A). Within the aggregate glands, expression of AgSp1 and AgSp2 is not significantly different (Figure 5B).

## Discussion

The aggregate glue spidroins AgSp1 and AgSp2 of *A. trifasciata* have coding sequences over 42 kb and 20 kb in length (respectively), both larger than the previous record-holding 19 kb aciniform prey-wrapping silk spidroin AcSp1 (Ayoub *et al.* (2013); Table 1). Both predicted proteins are structured as others in the family, containing repetitive central regions capped by conserved termini. The repetitive motifs are notably similar between the two spidroins, and predicted protein alignments of the repeat motifs from these two spidroins supports paralogous origins (Figure 3). Interestingly, AgSp2 is highly reduced in cobweb weavers (Supplementary Fig. S5). It is unknown if AgSp1 and AgSp2 interact with each other in any way to form the sticky capture glue, or what properties each imparts, particularly considering the differences between AgSp2 in orb web and cobweb weavers. However we are able to make inferences about the molecular function of aggregate spidroins based on extensive research of related spidroins in the silk family.

**Figure 6:**
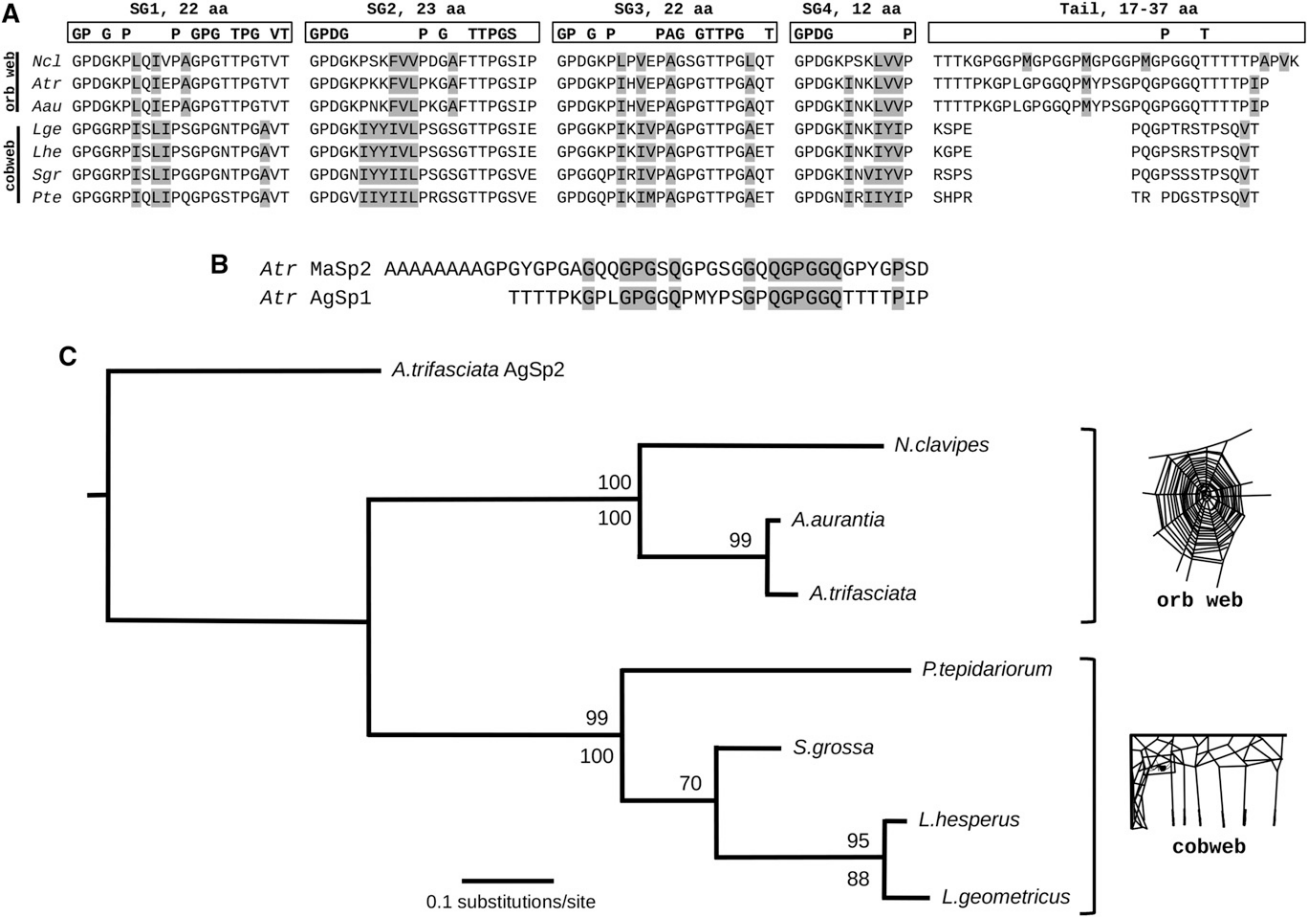
Repeat motif and phylogenetic comparisons for AgSp1 across species. (A) AgSp1 predicted amino acid repeat motifs for seven species: three orb web weavers (including repeat motif 2 from *A. trifasciata*) and four cobweb weavers. Each repeat consists of 4 subgroups (SG) with a conserved number of amino acids (aa) followed by a variable length tail. Conserved amino acid residues are within boxes. Gray highlight within species repeats indicates hydrophobic residues. (B) Aligned *A. trifasciata* major ampullate spidroin 2 (MaSp2) repeat (accession: AF350267.1) and AgSp1 repeat tail predicted proteins; gray highlight indicates shared amino acid residues. (C) Maximum likelihood tree with bootstrap support (values >50 shown) of repeat region encoding nucleotides (above node) and amino acid translation (below node; alignments in Supplementary Fig. S7). Tree was rooted using the repeat region of AgSp2 from *A. trifasciata*.

Orb web glue is much more extensible than cobweb glue (Sahni *et al.* 2011a), and, interestingly, the tail region of orb web AgSp1 repeats contains a higher proportion of short motifs similar to those that contribute to the extensibility of major ampullate (MA) and flagelliform (FLAG) silks (Gosline *et al.* 1999; Hayashi *et al.* 1999; Adrianos *et al.* 2013; Malay *et al.* 2017). Similar to MA and FLAG spidroins, orb weaver AgSp1 repeat tails contain GGQ groups, and share PGG, GPG, and QGP groups in common with MaSp2 (Figure 6). Furthermore, GPG motifs of minor ampullate spidroin proteins are significantly correlated with its extensibility (Vienneau-Hathaway *et al.* 2017). Not surprisingly, the stretches of poly-alanine that impart tensile strength to MaSp silk (Gosline *et al.* 1999) are lacking in the much weaker aggregate glue. The GPG and QGP regions are also found in the cobweb weaving species’ repeat motif tail regions, though the tails in these species are shorter and contain far fewer of these groups than their orb weaving counterparts (Figure 6A, Supplementary Figure S4). If the protein backbone is responsible for imparting extensibility (how far the glue stretches) reduction in the tail region may be a response to differing functional needs for cobweb prey capture. Cobweb weavers intercept ambulatory prey that accidentally trip their gumfoot lines at low speeds. In contrast, orb weavers capture highly mobile flying or saltatory prey, and the glue may need to be more extensible in order to remain in contact with higher velocity prey items.

Notably, AgSp2 of *A. trifasciata* has 60% more total glutamine than AgSp1 despite being only half the size. A quarter of glutamine in AgSp2 is part of QQ motifs located in high density between blocks of iterated repeats (Supplementary Fig. S2). Similar expansion regions are also found in pyriform spidroins (Chaw *et al.* 2017), and QQ motifs have been shown to allow self-aggregation of these silk proteins into fibers (Geurts *et al.* 2010). The coding sequence of AgSp2 is dramatically reduced in *Latrodectus* cobweb weavers, consisting of less than 2000 bp (Supplementary Fig. S5). Differences between orb weaving and cobweb weaving pyriform spidroins have also been noted, including a loss of the QQ motifs (Chaw *et al.* 2017).

**Figure 7:**
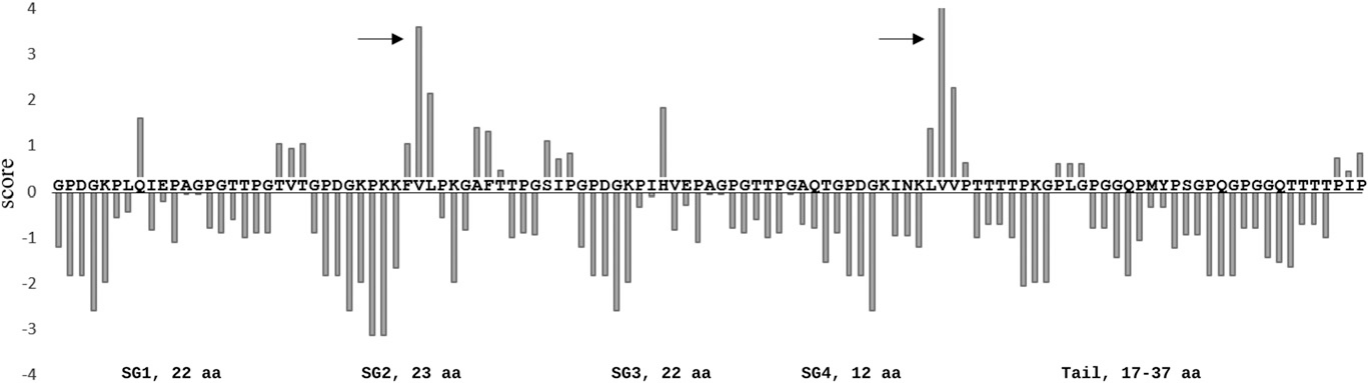
Hydrophobicity plot for repeat motif 2 of *A. trifasciata* AgSp1. Positive values indicate hydrophobicity. Arrows indicate peaks produced from triplets of hydrophobic amino acids (aa) in repeat subgroups (SG) 2 and 4 (see figure 6). Window size: 3.

Aggregate spider glue has been compared to the mucin family of proteins because of their glycosylated structure, viscoelastic properties (Shogren *et al.* 1989; Tillinghast *et al.* 1993; Choresh *et al.* 2009; Sahni *et al.* 2010), and highly repetitive central domains (Perez-Vilar and Hill 1999). Our study supports similarities of aggregate spider glue to this family of secretory glycoproteins, however distinct differences can also be noted. Particularly, mucins contain cysteine-rich regions, which are joined via disulfide bonds between protein monomers resulting in the mucous net barrier or gel-like characteristics common to mucins (Desseyn 2009; Bansil and Turner 2006). Mucins with more cysteine residues produce stronger barriers, protecting underlying epithelial layers (Gouyer *et al.* 2015). It has been hypothesized that the conserved 2-3 cysteine residues in each AgSp1 terminus may form disulfide bridges between silk monomers (Collin *et al.* 2016), however gel-forming mucins contain cys-rich domains between the glycosylated regions as well, which are lacking in AgSp1 and AgSp2. Groups of nonpolar and hydrophobic amino acids, however, could contribute to multimerizing the individual glycoprotein monomers. Furthermore, these residues may enhance the elasticity of the glue as they pull together and exclude water (Gosline 1978; Li *et al.* 2001; Bansil and Turner 2006), which is hypothesized to contribute to elasticity in hydration-dependent flagelliform capture spiral silk (Hayashi and Lewis 1998; Vollrath and Edmonds 1989; Bonthrone *et al.* 1992). Dense hydrophobic groupings (Figure 6A, highlight) in subgroups two and four of *A. trifasciata* AgSp1 RM2 correspond to distinct hydrophobic pockets in Kyte-Doolittle hydropathicity plots (Figure 7). These hydrophobic residues are conserved across AgSp1 and AgSp2 repeat motifs in *A. trifasciata* (Figure 3, highlight), and AgSp1 repeats in both orb web and cobweb weavers (Figure 6A, highlight). In contrast to extensibility, the glue needs to maintain elasticity, or the ability to resume its original shape, regardless of web type in order to retain prey that has contacted the threads.

The adhesive qualities of spider glue are due to protein glycosylation (Singla *et al.* 2018), and at least 80% of threonine residues in Argiope glues are likely O-glycosylated with mainly N-acetylgalactosamine (GalNAc)(Dreesbach *et al.* 1983; Tillinghast *et al.* 1993). The threonine residues of AgSp repeats are also flanked by glycine and proline, which is consistent with studies that show there is an increase in these residues around O-linked glycosylated threonines (Christlet and Veluraja 2001; Yoshida *et al.* 1997). Glycine is likely more prominent due to its small, non-interfering side-chains, whereas proline is hypothesized to play a structural role, kinking the protein in the form of *β*-turns. Furthermore, mucin-type glycosylated residues tend to occur in adjacent multiples, and this is consistent in the orb weaving AgSp repeats, where most threonines are directly adjacent to or within one residue from each other (Figure 3)(Christlet and Veluraja 2001). O-glycosylations prevent proteins from forming globules, and instead allow the protein to maintain an expanded conformation, as well as hydrating and solubilizing the protein (Shogren *et al.* 1989; Perez-Vilar and Hill 1999). Electron micrographs from earlier studies of spider glue proteins showed extended, filament-like molecular structure and allowed estimations of size from 450 to 1400 kD (Figure 4 in Tillinghast *et al.* (1993)); our estimations from the predicted AgSp1 (1,383 kD) and AgSp2 (676 kD) correspond to the upper and lower range values from the previous estimate.

There are differences in the percentages of amino acids that make up the AgSp1 repeats of orb webs and cobwebs (Figure 6A). Interestingly, repeats from both glues contain a similar number of O-glycosylation sites, however orb weaver glue has a higher percentage of threonine residues than cobweb glue, while cobweb glue contains more serine residues. Changes in humidity differentially affects orb and cobweb glue adhesion (Sahni *et al.* 2011a), and, in addition to low molecular weight compounds and salts, this contrast in function could be due to differences between the serine and threonine glycosidic linkages (Corzana *et al.* 2007). These linkages structure the surrounding water in different ways, forming water pockets and bridges between the sugar and protein backbone. Additionally, threonine is more readily glycosylated than serine (O’Connell and Tabak 1993), which could also contribute to the material differences observed between the two types of glues.

Spider silk has been notoriously difficult to synthetically produce due to the difficulty in replicating the transition from liquid protein dope to solid fiber, which occurs within a spider’s silk gland duct. Aggregate spider glue is extruded as an amorphous liquid that does not undergo the same processing, however the post-translational glycosylation may provide its own challenges for future synthesis. Encouragingly, there has been success using cloned and expressed glycosyltransferases to *in vitro* O-link GalNAc residues to synthetic peptides (Yoshida *et al.* 1997; Hagen *et al.* 1997). We have identified an N-acetylgalactosaminyltransferase (pp-GalNAc-T, BLAST e-value = 3.71e-174; accession#: MK138560) from *A. trifasciata*, which is part of the family of transferases that facilitates the addition of mucin-type, O-linked glycans by catalyzing the transfer of GalNAc from the sugar donor, UDP-GalNAc, to the hydroxyl groups of serine or threonine residues of the core protein, forming GalNAc-*α*-1-*O*-Ser/Thr. This specific GalNAc-T is differentially expressed in the aggregate glands compared to major ampullate gland and fat body tissues (q-value = 3.9e-8 and 7.8e-17, respectively). Furthermore, glue function does not seem to be limited by droplet size, as bolas spiders in the genus *Mastophora* produce droplets specialized for capturing fast-moving moths and are visibly large (*M. cornigera* droplets are ∼2.4 mm diameter, Hutchinson (1903); Figure 1C). *In vitro* glycosylation as well as the potential for bulk production are encouraging attributes for synthetic versions of aggregate spider glue.

## Conclusion

We used Illumina short reads aligned to long reads from Oxford Nanopore’s MinION to resolve aggregate spidroin encoding sequences AgSp1 and AgSp2 from orb weaving spider *Argiope trifasciata*. AgSp1 (42,270 bp) and AgSp2 (20,526 bp) are the largest spidroins currently described, each containing a large intron, and are of the most highly expressed genes within the aggregate glands. The predicted proteins consist of dominant central repetitive motifs and comparisons of AgSp1 repeat motifs across three orb web and four cobweb species suggests differences in the peptide backbone are related to observed functional differences of these two aggregate glue types.

As sequencing technology continues to improve, the genetics of complex biomaterials like silk spidroins will continue to be described. The similarities and differences between the orb web and cobweb glue repeat backbones are striking and provide useful insight into the relationship between form and function. Examining glue diversity from more distantly related species will allow an understanding of how differences in spider glue glycopeptides influence performance, contributing to the development of novel bio-inspired materials.
